# Clinical needs assessment to inform development of a new assay to detect antimalarial drugs in patient samples: A case study

**DOI:** 10.1371/journal.pgph.0002087

**Published:** 2023-08-24

**Authors:** Erin S. Coonahan, Chanaki Amaratunga, Carole A. Long, Joel Tarning

**Affiliations:** 1 Laboratory of Malaria and Vector Research, National Institute of Allergy and Infectious Diseases, National Institutes of Health, Rockville, Maryland, United States of America; 2 Mahidol Oxford Tropical Medicine Research Unit, Faculty of Tropical Medicine, Mahidol University, Bangkok, Thailand; 3 Centre for Tropical Medicine and Global Health, Nuffield Department of Medicine, University of Oxford, Oxford, United Kingdom; Menzies School of Health Research, AUSTRALIA

## Abstract

Point-of-care assays have greatly increased access to diagnostic information and improved healthcare outcomes globally, especially in the case of tropical diseases in rural settings. Increased recognition of the impact of these tools and increased funding, along with advances in technology have led to a surge in development of new assays. However, many new tools fail to fulfill their intended purpose due to a lack of clinical impact, operational feasibility, and input from envisioned operators. To be successful, they must fit into existing clinical decision-making models and be designed in collaboration with end users. We describe a case study of the development of a new low-cost sensor for antimalarial drugs, from initial planning through collection and incorporation of design feedback to final assay design. The assay uses an aptamer-based sensor to detect antimalarial drugs from patient samples for tracking antimalarial use in Southeast Asia, a region with a long history of emerging antimalarial drug resistance. Design and use-case input was collected from malaria control experts, researchers, and healthcare workers to develop target product profiles. Data was collected via surveys and in-person interviews during assay development and ultimately informed a change in assay format. This aptamer sensor platform can be easily adapted to detect other small molecule and protein targets and the design process described here can serve as a model for the development of effective new assays to improve access to healthcare technology.

## Introduction

*Plasmodium falciparum* malaria is responsible for more than 200 million human infections and over 600,000 deaths each year [1]. In recent decades, *P*. *falciparum* parasites in Southeast Asia have developed resistance to both the artemisinin component and the partner drugs in first line artemisinin-based combination therapies (ACTs), slowing progress towards malaria elimination [2–6]. A major concern is the potential spread of resistant parasites to the African continent where most of the world’s malaria cases occur and where first-line ACTs remain effective. Measuring drug content in patient samples and tablets is crucial for understanding patterns of drug use and identifying counterfeit therapies–both important factors contributing to resistance. Regulating medicine quality [7, 8] and access to approved therapies [9] is challenging. Current methods for detecting antimalarial drugs from tablets or patient samples require access to sophisticated laboratories capable of performing liquid chromatography-mass spectrometry (LC-MS)—the standard method for detecting antimalarial drug compounds [10–15]. LC-MS is highly sensitive but is time-consuming and often prohibitively expensive or operationally challenging as it requires shipment of samples to labs with the necessary expertise and equipment. A low-cost method of drug detection would improve access to drug use data and support efforts monitoring emerging antimalarial drug resistance.

In response to the spread of antimalarial drug resistance, the worldwide community of scientific organizations, governments, and funding bodies have increased efforts to study and contain drug-resistant parasites. In 2021, an estimated USD 3.5 billion was spent on malaria control and elimination worldwide and the World Health Organization (WHO) predicts this amount will need to exceed USD 10 billion annually to implement successful elimination strategies [1]. Malaria elimination will require a concerted effort from numerous countries and stakeholders and investment in effective interventions.

Point-of-care diagnostics have proven to be an especially valuable tool in the fight against malaria. Malaria rapid diagnostic tests (RDTs) have been widely adopted with more than a 10-fold increase in worldwide sales over the last 10 years and an estimated 413 million sold in 2021 [1]. Point-of-care tests are so named because they provide quick data to inform clinical decisions where care is administered without requiring access to specialized or centralized laboratories. In comparison, the term rapid assay refers to a simple, often lateral flow, single use assay that provides a quick output not necessarily at the point-of-care. New point-of-care and rapid assays have been designed to improve access to research, diagnosis, and treatment, such as tests to screen for Glucose-6-phosphate dehydrogenase (G6PD) deficiency and platforms for other infectious diseases such as human immunodeficiency virus (HIV) and tuberculosis [16–18]. However, many new tools are never adopted due to a lack of clinical need or operational feasibility [19, 20]. To ensure that a newly proposed assay can be successfully adopted, it must fill a clear need, meet technical requirements, and if it is a clinical tool, it must fit within clinical workstreams [21]. Clinical needs assessments are essential to inform the design of new tools, especially in the case of new tools for global health where funding for market research is limited and technical barriers are abundant [22].

Here, we describe a case study based on the development of a new assay to aid the study of antimalarial drug resistance, beginning with idea conception, pausing development to perform a clinical needs assessment, and incorporating feedback into a target product profile to guide assay design. We envisioned developing a low-cost, field-based assay to detect slow-clearing ACT partner compounds from patient samples to track drug use and treatment failures in Southeast Asia to inform future treatment decisions at the point-of-care.

## Materials and methods

### Initial assay planning/development

The idea of a low-cost, point-of-care assay to detect slow-clearing ACT partner compounds from patient blood samples was envisioned through conversations with malaria researchers at the National Institutes of Health, USA. The initial proposed use-case was to characterize recent treatment failures to inform future treatment in a region with high rates of dihydroartemisinin-piperaquine failure due to parasite resistance. Slow-clearing ACT partner compounds such as piperaquine remain at detectable levels in blood samples for weeks after treatment [23–26]. Detection of these compounds in a currently infected patient could suggest recent treatment, possible treatment failure and parasite recrudescence (if a curative dose was taken), triggering future treatment with an alternative drug. After initial research to understand technical feasibility and design of the sensor, it was decided that clinical need and specifications of the assay should be further evaluated. Technical development was paused, and the following data collection was performed.

To understand the utility of the envisioned assay and define desired design specifications, we planned a study to collect feedback from potential end users and those familiar with the assay’s intended environment of use. Three groups of stakeholders were identified: 1) academic researchers in malaria and public health fields to gather broader feedback from peers, 2) malaria clinicians and researchers based at clinic sites to understand the desired assay characteristics from envisioned end-users, and 3) government officials and funding agencies believed to be decision makers about the adoption of new tools. Feedback was collected through an online survey and in-person interviews to develop target product profiles [27].

### Selection of interviewees/survey recipients

Participants were identified via purposive sampling and were initially contacted via email. Survey participants received the questionnaire via email and interviews were arranged to be conducted in person. To be considered for the online survey, participants had known relevant experience in the fields of malaria biology, drug resistance, or malaria control and public health efforts. The online questionnaire was designed to collect structured input from geographically diverse stakeholders. It was sent to 30 individuals working in the field of malaria control and research; 18 participants responded (Table 1).

**Table 1:** Description of online survey respondents and in-person interviewees. Most respondents reported working across multiple countries with a higher proportion based in Southeast Asia. All survey respondents belonged to the academic research group while interview participants were split across 3 stakeholder groups.

Stakeholder group	Number	Countries of work
*Survey respondents*		
Academic researcher in malaria or public health field	18	Brazil (3), Cambodia (4), Colombia (1), Kenya (2), Laos (1), Mali (1), Myanmar (4), Peru (1), Senegal (3), Switzerland (2), Thailand (6), Uganda (1), United Kingdom (1), United States (2), Zambia (1)
*Interview participants*		
Academic researcher in malaria or public health field	9	Cambodia (5), Indonesia (1), Laos (2), Malawi (1), Mozambique (1), Myanmar (2), South Africa (1), Thailand (7), Vietnam (3), Zimbabwe (1)
Employee in government or funding agency	11	Cambodia (9), Thailand (1), Myanmar (2), Vietnam (1), Angola (1)
Field researchers/ healthcare workers based in malaria clinics	10	Cambodia (3), Myanmar (8), Thailand (8), Vietnam (2)

For interviews, a semi-structured in-depth interview guide was designed to add context to the responses from the survey and reach front line healthcare workers and stakeholders at funding and governmental organizations who we were not easily reachable via online survey. Interviewees were required to have known experience working in the field of malaria control or treatment in Southeast Asia. They were selected to cover a range of identified stakeholders including members of funding bodies and governmental agencies, clinicians and researchers primarily based in malaria clinics with familiarity with the end-use setting, and academic researchers more remotely based who may have a broader perspective on some areas of questioning but less direct knowledge of the assay’s intended environment of use.

30 in-person interviews were conducted between March-May 2016. Two individuals contacted for interview did not participate due to scheduling conflicts. 9 interviewees were identified as malaria researchers, 11 as people working at funding/development or government agencies, and 10 as field-based clinicians. All interviewees reported working in Thailand or Cambodia, where interviews were conducted, and several also had relevant work exposure in other countries. Affiliation and country of work for all participants are listed in Table 1.

Desired sample sizes were based on literature review estimates of participants required to reach thematic saturation in studies across diverse fields of qualitative research. Based on this review, we estimate that >80–90% of concepts should be reported by the first 10–12 participants [28–30]. Taking into account the expected prevalence of a theme within the study population, and using a conservative population theme prevalence of 50%, we can estimate that a sample size of 8 participants in each stakeholder group and modality of data collection would result in reporting of at least 3 theme instances at 80% confidence using a previously published model [31]. These data and models provided context for approximate sample size estimates, which were then reassessed for thematic saturation during data collection to confirm adequate size. While the survey allowed for the collection of quantitative data, sample sizes were not intended to provide sufficient power for statistical analysis or to draw conclusions comparing assay design for specific geographies, rather the goal was to collect more diverse opinions on assay design and utility from academic researchers in the field beyond authors and close contacts.

#### Online survey

An online questionnaire was developed using Survey Monkey’s web-based platform and optimized through in-person pre-testing and pilot testing. During pre-testing, selected persons with relevant experience were asked to verbalize responses and areas of confusion while completing the survey under supervision. This provided feedback to clarify survey design before pilot testing. Participants of in-person pre-testing were excluded from the final questionnaire data set. During pilot testing, the survey was sent first to a subset (n = 4) of the potential respondents. Once these results were evaluated for clarity, the questionnaire was sent to the remaining members of the study respondents (copy of online questionnaire in S1 File).

#### Interviews

In-depth, semi-structured interviews were conducted by ESC in Bangkok and Mae Sot, Thailand and Phnom Penh, Cambodia. Interviews were conducted in-person and in English with translation help when necessary. They ranged from 20 to 60 minutes and were conducted at the interviewee’s place of work. The conversations were transcribed by the interviewer during interviews and transcriptions were later coded by themes (copy of semi-structured interview guide in S2 File).

### Data analysis

We planned a mixed methods study in which quantitative and qualitative data were collected simultaneously and in which qualitative interview data was intended to provide context to survey responses and explore new content areas by reaching new stakeholder groups. Responses from the survey and themes from the in-person interviews were sorted by frequency to assess study respondent’s opinions of the utility of a low-cost drug detection assay, as well as suggested operational characteristics. All survey respondents belonged to the stakeholder group identified as academic researchers. Survey data was grouped into the following topics derived from survey questions: suggested applications of the assay, preferred sample type, preferred drug targets, assay cost, quantitative (numerical or range) vs qualitative (yes/no) assay format, and other suggested assays.

Interviews were sorted by the interviewee’s role/affiliation to reveal any differences in opinion stemming from participant experience and workplace exposure. Themes from interview transcriptions were identified following the interview guide and emerging themes were organized through a content analysis by ESC with coding verification and guidance from the Mahidol Oxford Tropical Medicine Research Unit (MORU) Bioethics and Public Engagement department. Themes were sorted using a deductive approach and data was organized in Microsoft Excel. Coded responses were sorted by frequency and by stakeholder group of the interviewee. The qualitative data collected in the interviews was explored to identify new themes across stakeholder groups. Interview data from participants in the researcher stakeholder group also provided context for interpretation of survey responses.

### Ethical approval

This study was submitted to and approved by the Oxford Tropical Research Ethics Committee (OxTREC). The online survey and in-person interviews were deemed market research constituting a service review with the aim of improving the current level of care and exempt from committee review. Objectives of the online survey were stated at the beginning, participation was voluntary and anonymous except when participants provided contact information for follow up, which was explicitly stated. During in-person interviews, objectives were stated at the beginning and permission to conduct the interview was collected verbally.

## Results

### Potential applications of the assay

Of the 18 survey participants, 17 reported working in a setting where rapid diagnostics or point-of-care assays are used and 10 reported having a need for a point-of-care assay to detect antimalarial drugs from human samples in the last year. It should be noted that point-of-care as referred to by authors suggests a setting where clinical decisions are being made and care is administered. However, as described below, some of the most cited use-cases for a drug detection assay would not require results at the point-of-care, suggesting that there may have been some inflation in reported interest in a point-of-care assay based on variation in the respondent’s definition of “point-of-care.”

Among survey respondents, the most suggested applications for a drug detection assay were to survey drug use in a region, for inclusion/exclusion to a research protocol, and to monitor adherence during mass drug administration campaigns (Table 2). Answers were selected from a pre-specified list or written in as optional free text answers. These responses suggested that survey respondents envisioned a drug detection assay as a research tool rather than a point-of-care clinical assay.

**Table 2:** Utility of a field-based sensor to detect antimalarial drugs reported by online survey. Survey respondents answered questions about the need of a point-of-care or field-based assay for antimalarial drug detection.

Question	Answer Options	% (N)
Does your work involve settings where rapid diagnostics or point-of-care assays are used?	Yes	94.4 (17)
	No	5.6 (1)
In the past year, have you had a need for a point-of-care assay to detect antimalarial drugs from patient samples?	Yes	55.6 (10)
	No	44.4 (8)
What do you think would be the most relevant applications for a field-based drug detection assay? Please select all that apply.	Surveillance of drug use in a region	77.8 (14)
	Inclusion/exclusion to a research protocol	72.2 (13)
	Monitoring adherence during mass drug administration campaigns	72.2 (13)
	Measuring drug levels as an indicator of drug absorption and treatment efficacy	66.7 (12)
	Detecting falsified/substandard drugs (from crushed tablets)	61.1 (11)
	Assessing adherence to primaquine for *P*. *vivax* treatment	5.6% (1)
	To assess drug levels in setting of serious adverse events	5.6% (1)

**Fig 1:**
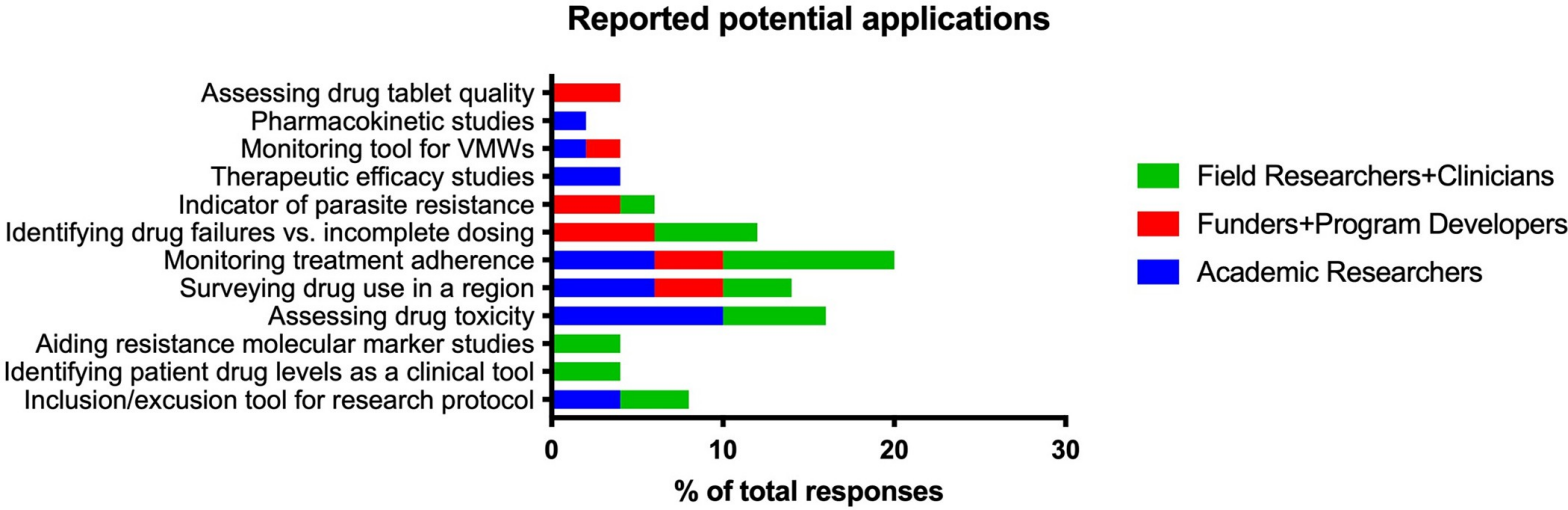
Reported potential assay applications from in-person interviews. Most cited applications reported in the interviews across stakeholder groups follows that of the researchers’ responses in the online survey (Table 2). The most common responses propose the use of this assay as a research tool that could operate in a laboratory to collect data on drug use and treatment adherence.

When asked how they might use a rapid, low-cost drug detection assay, interviewees identified 12 potential applications. They also most commonly cited applications for research purposes including monitoring adherence during mass drug administration, assessing drug toxicity, and surveying drug use in the region. Although, several of these reported applications such as assessing drug toxicity and measuring patient drug levels in the clinic or for mass drug administration monitoring could benefit from a point-of-care assay format. (Fig 1).

**Fig 2:**
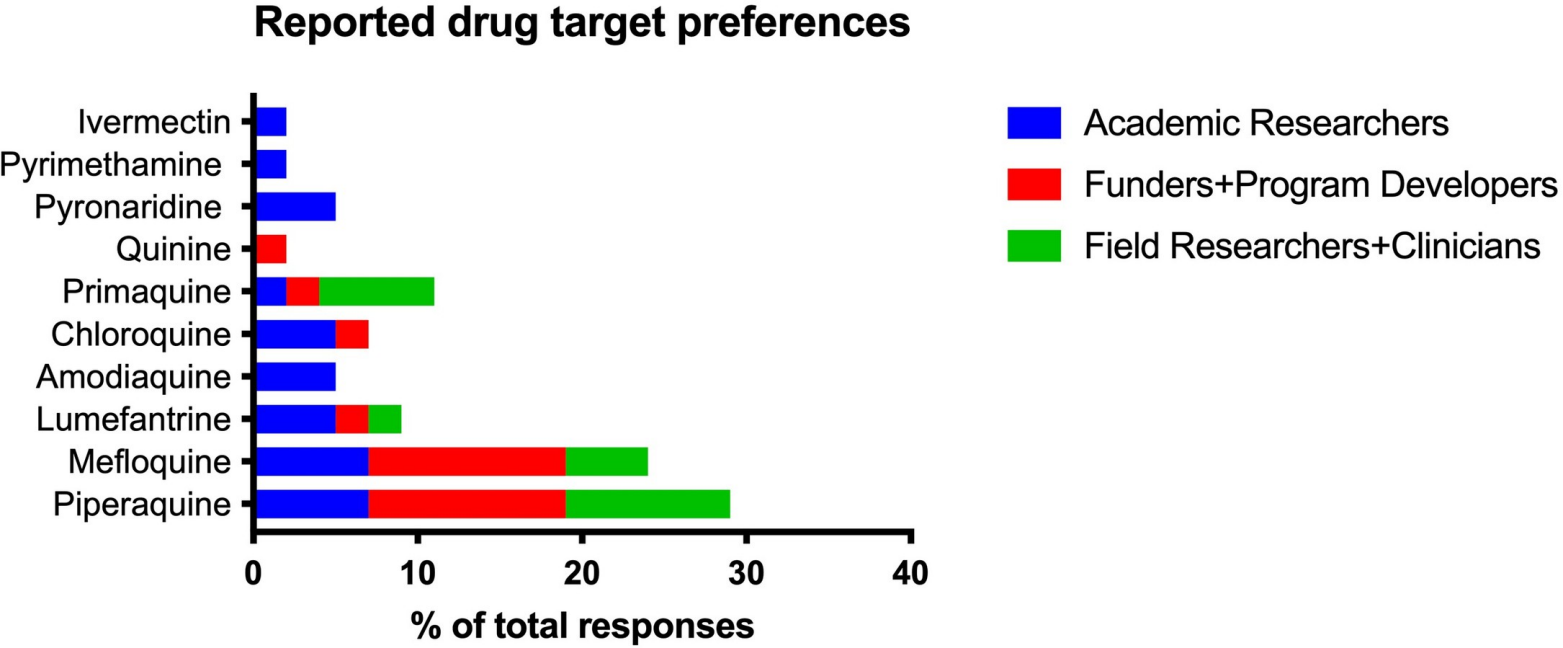
Target drug preferences reported during in-person interviews. Sorted responses (N = 30) report a preference for piperaquine and mefloquine which reflects the geographic distribution of interviewees who work primarily in Southeast Asia where piperaquine and mefloquine were the most recently used ACT partner drugs.

Regarding the recommended drugs to target for detection, survey respondents most commonly selected piperaquine, lumefantrine, artemisinin-derivatives, primaquine, and mefloquine. These target drug references were found to align with first-line therapies in the country where the respondent worked (Table 3). The preferred drug targets reported in interviews also corresponded with the participant’s region of work for the most part, while those in the academic researcher group cited a wider range of drug targets. This resulted in a preference for piperaquine and mefloquine detection in the in-person interviews as they were mostly conducted in Thailand and Cambodia where most recent first-line therapies were dihydroartemisinin-piperaquine and artesunate-mefloquine (Fig 2).

**Table 3:** Target drug preferences reported by online survey. Participants (N = 18) were able to select multiple target drug preferences. Reported target drugs were found to align with first line therapies in the participant’s listed countries of work.

Question	Answer Options	% (N)
Based on the applications you envision, what drugs do you think would be most useful to detect in a point-of-care setting? Please select all that apply.	Piperaquine	77.8 (14)
	Lumefantrine	77.8 (14)
	Primaquine	66.7 (12)
	Artemisinin/artemisinin derivatives	66.7 (12)
	Mefloquine	55.6 (10)
	Amodiaquine	50 (9)
	Sulfadoxine/pyrimethamine	33.3 (6)
	Chloroquine	27.8 (5)
	Other (please specify)	0.0 (0)

### Desired operational characteristics of the assay

**Fig 3:**
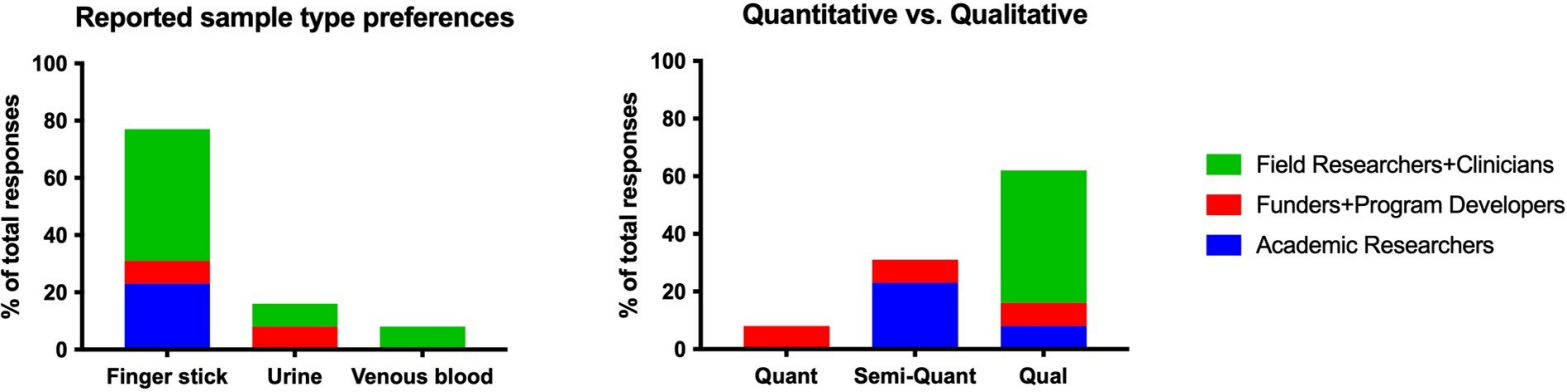
Preferred sample type and assay readout reported during in-person interviews. Finger stick samples were preferred, though conversations highlighted that it would be ideal if venous blood could be used interchangeably. A preference for a qualitative readout was driven primarily by field researchers and clinicians (N = 30).

Regarding the operational characteristics of the assay, survey respondents ranked four possible sample types, from most to least practical, with finger stick blood recorded as the most practical and venous blood as the least practical (Table 4). Similarly, most interviewees across stakeholder groups reported that a finger stick sample would be most useful (Fig 3). However, interviews highlighted a scenario where the assay could ideally provide accurate readings for both finger stick and venous blood. This would negate the need for an additional fingerstick in the case where a venous draw had already been collected.

**Table 4:** Desired operational characteristics reported by online survey. Participants (N = 18) reported desired operational characteristics such as cost, sample type, and readout. Participants were able to skip questions.

Question	Answer Options	% (N)
How much could this assay cost in order for it to be feasible for use?	Less than 1 USD	66.7% (12)
	Between 1–5 USD	22.2% (4)
	More than 5 USD	11.1% (2)
	Other (please specify)	0% (0)
What sample type would be the easiest to use in this point-of-care assay? Please rank most practical (1) to least practical (4).	Sample Type	Average Rank (N = 17)
	Finger stick blood	1.8
	Saliva	1.9
	Urine	2.9
	Venous blood	3.3
On a scale of 0–100, how important is it that the assay be semi-quantitative (provide a range of concentrations) versus qualitative (appearance of line indicates some drug present).	0 = semi-quantitative 100 = qualitative.	Average score from 0–100: 54 (N = 13)

Survey respondents were asked about assay cost and 67% reported that the assay should cost no more than USD 1, 22% suggested it could cost up to USD 5 and still be feasible for use. Survey respondents suggested it would be moderately important (54 on a scale of 0–100) that the assay provide a semi-quantitative (numerical range) versus qualitative (yes/no) output based on envisioned applications. (Table 4). It should be noted that the survey responses represent the opinions of members of the malaria researcher stakeholder group. In discussions of readout format in the interviews, several healthcare workers and those familiar with clinic work in rural malaria-endemic settings explained a preference for a qualitative assay due to ease of interpretation over semi-quantitative or quantitative in that setting. Fig 3 shows this preference for a qualitative assay, driven by interviewees identified as field researchers and clinicians. However, interviews also highlighted the case of a lab-based assay used by malaria researchers, in which a quantitative readout was preferred since this information would be useful for certain applications and the environment would allow for more complex interpretation of results.

### Assessing clinical need for other rapid assays

To assess clinical needs for assays aside from the drug detection assay, participants of the online survey and interviews were asked which rapid assays are most needed to improve malaria elimination efforts right now. Survey respondents most commonly cited a higher sensitivity rapid test to detect both *P*. *falciparum* and *P*. *vivax* as their greatest need for a new assay, followed by a rapid test to determine G6PD deficiency prior to primaquine administration. Other cited assays included test for gametocyte detection to identify transmissible reservoirs even if asexual parasitemia had cleared and a rapid test to identify substandard drugs, which could be deployed in a pharmacy or QC lab.

In interviews, two assays were mentioned—a higher sensitivity malaria RDT and a test for G6PD deficiency, similar to survey responses. One interviewee in the funders and program development stakeholder group stated “everyone is now focused on radical vivax cure due to a big push from the WHO” which would require combined treatment of blood and liver stage parasites and a reliable point-of-care G6PD assay prior to primaquine administration. Two interviewees of the field researchers and healthcare workers group stated that the readout of the current G6PD assay available to them has a “very faint positive line” and is too difficult to reliably interpret. Members of this group also explained a need for a higher sensitivity *P*. *vivax* detection assay. One member explained that they are not able to detect *P*. *vivax* cases in communities with an estimated 20–30% infection rate due to sensitivity of current assays. Research and development to create both of these most cited tools is currently underway by various companies and research programs and this line of questioning helps confirm the need for such technology.

### Target product profiles (TPPs) for the assay

Based on the information collected via the online survey and the in-person interviews, an interest in two different formats for a drug detection tool was reported and thus two target product profiles were developed. Surprisingly, there was considerable interest in a version of this tool primarily as a research assay. Most cited applications included surveying drug use in a region, monitoring success of mass drug administration campaigns, and for inclusion/exclusion to research protocols—none of which require the assay to provide a point-of-care readout. An affordable assay that could be used in small labs in Southeast Asia requiring only standard lab equipment would allow researchers to collect information on drug use in a timely and cost-effective manner as compared to shipping samples for (LC-MS) analysis. This lab-based assay would ideally be quantitative or semi-quantitative and detect multiple drugs to increase its utility for a variety of applications. The consumables need to be readily accessible and ideally priced at around 1 USD per sample. This cost would be approximately 20–30 times lower than the expense of academic collaborators’ LC-MS services [15], which are already considerably more affordable than those provided by contract research organizations. The assay could require access to standard bench top equipment, though this may limit its accessibility. The details of this target product profile are shown in Table 5.

**Table 5:** Target product profiles (TPPs) drafted using input from online survey and in-person interviews. TPP1 describes a lab-based tool with quantitative readout for research purposes. TPP2 describes a rapid, point-of-care format, as was initially envisioned. Based on reported interest in applications of the proposed assay, development focused on TPP1. Desired assay sensitivity is reported in days post treatment as drug concentrations will vary with the pharmacokinetic properties of drug targets.

**Target Product Profile 1: Inexpensive lab-based assay**			
**Suggested use cases:** survey drug use in a region, for inclusion/exclusion to a research protocol, monitor adherence during MDA campaigns, assess medicine quality, confirm dosing/absorption to identify treatment failures			
**Parameter**	**Ideal**	**Acceptable**	**Comments**
**Cost**	< 1 USD	1–5 USD	This refers to the cost of consumables. The assay may require access to bench top equipment.
**Sample type**	Finger stick or venous blood (or dissolved tablets)	Plasma or serum from finger stick or venous blood (or dissolved tablets)	Preference for smallest possible blood volume. Preference for unprocessed blood but centrifugation to isolate plasma accepted.
**Drug target**	All partner drugs in use in the region	One first-line partner drug	Depends on region and desired application.
**Quantitative vs. Qualitative**	Quantitative	Semi-quantitative	
**Sensitivity**	3–30 days	3–7 days	
**Access to equipment**	Portable and inexpensive fluorimeter or spectrophotometer. All consumables included in kit.	Benchtop fluorimeter or spectrophotometer, may require access to standard lab consumables such as well-plates.	
**Target Product Profile 2: Point-of-care clinical assay**			
**Suggested use cases:** Assess drug toxicity, identify prior treatment and dosing, replace directly observed therapy in MDA campaigns			
**Parameter**	**Ideal**	**Acceptable**	**Comments**
**Cost**	< 1 USD	1–5 USD	Refers to entire kit
**Sample type**	Finger stick and venous blood	Finger stick blood	Ideally would work similarly with finger stick and venous blood
**Drug target**	All partner drugs in use in the region	One first-line partner drug	
**Quantitative vs. Qualitative**	Qualitative	Qualitative	Qualitative was reportedly preferred over quantitative for field-based point of care settings
**Sensitivity**	3–30 days	3–7 days	30 days after treatment would identify more people if using for surveillance and could identify failed treatments
**Access to equipment**	No benchtop equipment required, all components packaged together	No benchtop equipment required, all components packaged together	

The second target product profile describes a clinical tool for scenarios where it would be useful to have data at the point-of-care. However, there was reportedly little interest in an assay that could detect drug levels as an indicator of previous treatment to inform future treatment as initially envisioned. Interview discussions revealed that this initially proposed model, in which healthcare workers assess current drug levels to determine next treatment was unrealistic, in part because of the complexity of the treatment decision and in part because often only one first-line antimalarial therapy is readily available. Thus, the first target product profile describing a lab-based research tool was prioritized. However, based on our data, if a point-of-care drug detection assay were developed, it should have a qualitative readout, should cost no more than 5 USD per test, including all necessary components, and run-on small volumes of finger stick or venous blood (Table 5).

### Final assay design and validation

Following the described clinical needs assessment, the plan for assay development was adjusted to focus on the product described in TPP1 –a lab-based tool to enable local researchers to collect data on drug use and adherence in the region. To achieve this, a fluorescent aptamer-based sensor was developed. The final sensor detects both piperaquine and mefloquine, based on intended use in Southeast Asia. Assay components cost less than 0.25 USD per sample and only require access to a benchtop fluorimeter. The sensor function was validated in patient samples against LC-MS gold standard drug detection methods. The sensor is able to detect piperaquine from patient samples with a limit of detection of 2 ng/mL and mefloquine at 8ng/mL, allowing for detection up to several weeks after drug administration. The sensor was further adapted to detect piperaquine and mefloquine from crushed tablets to identify falsified/substandard tablets. The full technical design, detailed sensor development methods, and characterization of the assay performance are reported elsewhere [32].

## Discussion and conclusions

The creation of new point-of-care tools is often motivated by technological advances and research ideas rather than a defined clinical need [33, 34]. This approach can result in misguided design and the use of resources to develop tools that will not be adopted in the clinic as they were envisioned in the lab. In this study, initial attempts to develop an ACT partner drug detection assay began after discussions with peers in the malaria research field. However, during early development, a lack of clarity regarding the operational characteristics of the assay and the feasibility of its envisioned use led to a pause in assay development to prioritize understanding the utility of such a tool through a clinical needs assessment. Feedback from the clinical needs assessment informed a shift in the envisioned assay from a point-of-care clinical tool to a lab-based research assay.

Our study highlights a common misconception that the preferred format for new assays with global health applications is always a rapid, point-of-care test. A clinical needs assessment determined that our initial assay idea, a point-of-care drug detection tool signaling previous treatment failures to inform future treatment, was likely not feasible. However, study participants reported an interest in an affordable lab-based drug detection assay. An alternative method to LC-MS for measuring drug levels from patient samples that could be performed by researchers in their own labs in Thailand and Cambodia would allow them to expand research capabilities to study drug use, regulation, and patterns that contribute to emerging resistance. Prior to this survey, a lab-based assay platform had not been considered. New, accessible laboratory technology could empower local researchers to collect and analyze their data without relying on collaborations and access to central laboratories.

The feedback collected in this survey suggested that a point-of-care drug detection assay was less helpful than the authors had anticipated. This is especially true in settings where healthcare workers have access to a single first-line therapy mandated by country guidelines, as is currently the case across Southeast Asia where the majority of data was collected. However, as parasite resistance to artemisinin derivatives and partner drugs in ACTs continues to spread and treatment guidelines shift, countries may soon adopt a multiple first-line therapy (MFT) approach to malaria treatment [35–37]. In this case, it could be important to ascertain a patient’s treatment history from a drug detection assay to inform a future drug choice at the point-of-care. As disease landscapes change, technological needs also shift, and continued surveying of assay requirements is required. Perhaps in the setting of MFT for malaria control, a point-of-care assay format would be prioritized, and its design should be revisited.

The conclusions of this survey are somewhat limited by the number and geography of participants. The online survey was sent to 30 individuals and we received 18 responses which was limited based on feasibility of our purposive sample model and response rates. However, we feel this number was sufficient to achieve our goal of including opinions from diverse geographies, and suggesting the influence of geography on certain responses. Drawing any conclusions specific to the various geographies represented in the survey would require larger sample sizes. Nevertheless, including participants based in Africa helped solidify the conclusions that most researchers are interested in detecting the drugs that are first line therapies in their research setting. There were no notable differences between those who responded to the online survey and those who did not. Most participants of both the survey and interviews were based in Southeast Asia. For the purpose and scope of this research, the data collected improved understanding of realistic assay applications and operational characteristics necessary for use in Southeast Asia. Southeast Asia was our envisioned environment of use as this is where malaria treatment failures are occurring and where containment of resistant parasites is of the utmost importance.

This research could be impacted by several types of bias. One form being desirability reporting bias which could skew the results towards a more positive representation of the utility of the assay. It is likely that participants of the online survey and in-person interviews felt encouraged to report a need for the assay based simply on the fact that they were being asked by a researcher who was considering developing it. To try to avoid this, the survey responses were kept anonymous except in the instance where respondents provided their contact information to receive a report of the results (which was made clear at the beginning of the survey). In-person interviews were not anonymous (although respondents were informed that anonymity would be maintained during reporting) and were more likely influenced by this type of reporting bias. The interviewer made extra effort to establish at the beginning of each interview that the goal was to determine whether such a tool would be useful and gather honest feedback to inform assay development. However, to reduce bias, perhaps a better method would be to have the interviews conducted by a third person who was not invested in assay development. Similarly, question order bias could skew some responses. Prior lines of questioning may have influenced respondents to envision certain applications or operational characteristics. Also, the data could be skewed by selection bias in which those who agreed to participate in the survey/interviews may have already had more interest in the assay than those who declined.

In conclusion, collecting feedback to assess utility and required operational characteristics must be performed before time and resources are used to develop new point-of care tools and to adapt them to changing medical needs. Such research is especially important before the development of new tools for low-resource settings where there is an incorrect opinion that a general lack of access to technology will result in the more liberal adoption of new tools. In fact, tools designed for rural or low-resource settings often encounter unpredictable barriers to use. Here, we report an example where a clinical needs assessment informed the design of a new sensor to detect antimalarial drugs from patient samples. Data collected in this study resulted in a change in envisioned assay format from a rapid point-of-care test to a lab-based tool to improve local researcher’s ability to collect data on drug use and regulation in Southeast Asia. The methods of data collection described can be applied before design of other novel assays for global health applications and the resulting aptamer assay platform can be adopted to detect a variety of new targets.

## Supporting information

S1 File Copy of online questionnaire. (PDF)

S2 File Copy of semi-structured interview guide. (DOCX)
