# Supplementary material for: Clinical needs assessment to inform development of a new assay to detect antimalarial drugs in patient samples: A case study
Source: PLOS Glob Public Health. 2023 Aug 24;3(8):e0002087. doi: 10.1371/journal.pgph.0002087 (PMC10449106; doi:10.1371/journal.pgph.0002087)
Supplement: S1 File — (PDF) [file pgph.0002087.s001.pdf]

Point-of-Care Drug Detection Assay

Introduction:

We appreciate your consideration of this request. The goal of this survey is to collect feedback for the development of a point-of-care assay to detect antimalarial drugs from patient samples. It is being conducted as part of a PhD research project under supervision from the University of Oxford and the National Institutes of Health, USA.

The envisioned assay aims to detect the slow-clearing partner drugs of artemisinin based combination therapies which remain at detectable levels in the blood up to several weeks after treatment. It will provide a simple colored readout on paper as depicted in the diagram below. It will be inexpensive and feasible for use in rural malaria-endemic settings.

We seek your opinion on the realistic applications of the assay and design components that could improve its utility. This is a voluntary research survey and your answers will remain anonymous unless you provide any contact information. **It should take 7-10 minutes to complete** and your feedback will be used to inform design decisions about the assay.

1. What best describes the organization where you work/study/volunteer?

- ○ Funding and/or program development organization
- ○ University or research institute
- ○ Clinic/place where medical care is administered
- ○ Other (please specify)

33%

Prev Next

2. Which country/countries do you work in? Please select all that apply.

- [ ] Australia
- [ ] Bangladesh
- [ ] Brazil
- [ ] Cambodia
- [ ] China
- [ ] Colombia
- [ ] Germany
- [ ] India
- [ ] Indonesia
- [ ] Kenya
- [ ] Laos
- [ ] Malasia
- [ ] Mali
- [ ] Myanmar
- [ ] Nepal
- [ ] Papua New Guinea
- [ ] Peru
- [ ] Solomon Islands
- [ ] Switzerland
- [ ] Thailand
- [ ] United Kingdom
- [ ] United States
- [ ] Vietnam
- [ ] Other (please specify)

3. What antimalarial drugs are relevant to your research goals/clinical responsibilities? Please select all that apply.

- [ ] Artemisinin/artemisinin derivatives
- [ ] Piperaquine
- [ ] Mefloquine
- [ ] Amodiaquine
- [ ] Lumefantrine
- [ ] Chloroquine
- [ ] Primaquine
- [ ] Sulfadoxine/pyrimethamine
- [ ] Other (please specify)

4. Does your work involve settings where rapid diagnostics or point-of-care assays are used?

- ( ) Yes
- ( ) No

5. In the past year, have you had a need for a point-of-care assay to detect antimalarial drugs from patient samples?

- ( ) Yes
- ( ) No

6. What do you think would be the most relevant applications for a field-based drug detection assay? Please select all that apply.

- [ ] Detecting previous treatment failures
- [ ] Inclusion/exclusion to a research protocol
- [ ] Surveillance of drug use in a region
- [ ] Measuring drug levels as an indicator of drug absorption and treatment efficacy
- [ ] Monitoring compliance during mass drug administration campaigns
- [ ] Detecting fake/substandard drugs (from crushed tablets)
- [ ] Other (please specify)

7. Based on the applications you envision, what drugs do you think would be most useful to detect in a point-of-care setting? Please select all that apply.

- [ ] Artemisinin/artemisinin derivatives
- [ ] Piperaquine
- [ ] Mefloquine
- [ ] Amodiaquine
- [ ] Lumefantrine
- [ ] Chloroquine
- [ ] Primaquine
- [ ] Sulfadoxine/pyrimethamine
- [ ] Other (please specify)

8. Please briefly explain why you chose these:

9. Is there a scenario where it would be useful to detect just one drug?

- ( ) Yes
- ( ) No

10. If yes, which drug?

- ( ) Artemisinin/artemisinin derivatives
- ( ) Piperaquine
- ( ) Mefloquine
- ( ) Amodiaquine
- ( ) Lumefantrine
- ( ) Chloroquine
- ( ) Primaquine
- ( ) Sulfadoxine/pyrimethamine
- ( ) Other (please specify)

11. Is there a scenario where you would need to be able to detect more than one drug in order to be useful?

- ○ Yes
- ○ No

12. If yes, which drugs? Please select all that apply.

- ☐ Artemisinin/artemisinin derivatives
- ☐ Piperaquine
- ☐ Mefloquine
- ☐ Amodiaquine
- ☐ Lumefantrine
- ☐ Chloroquine
- ☐ Primaquine
- ☐ Sulfadoxine/pyrimethamine
- ☐ Other (please specify)

13. How important is it that the assay be semi-quantitative (provide a range of concentrations) versus qualitative (appearance of line indicates some drug present).

Not important. A qualitative assay would be very useful.	Somewhat important. Quantitative results would be helpful but not necessary.	Very Important. Quantitative results are necessary. A qualitative assay would not be useful.

14. What sample type would be the easiest to use in this point-of-care assay?

Please rank most practical (1) to least practical (4).

- Venous blood
- Finger stick blood
- Saliva
- Urine

83%

Prev | Next

15. How much could this assay cost in order for it to be feasible for use?

- ○ Less than $1
- ○ Between $1 and $5
- ○ More than $5
- ○ Other (please specify)

16. Please rank the most needed new point-of-care assays for malaria control and elimination. (#1 = most urgently needed, #5 = least urgently needed)

- G6PD deficiency
- Identifying substandard drugs
- Detecting low-density malaria infections
- Antimalarial drug detection in human samples
- Identifying antimalarial drug resistance markers

17. If not listed above, is there another new point-of-care assay that could improve progress towards malaria control and elimination?

18. Please include any further feedback you are willing to provide. Specifically, how you think a point-of-care assay for antimalarial drug detection could be useful, necessary components in order for it to be useful, and/or any envisioned issues with this assay idea.

19. Are you willing to be contacted if we have any follow-up questions?

- ○ No
- ○ Yes. Please provide your email address:

20. Would you like to receive a summary of the aggregated results of this survey?

- ○ No
- ○ Yes. Please provide your email address:

100%

Prev Done
