# Supplementary material for: Clinical needs assessment to inform development of a new assay to detect antimalarial drugs in patient samples: A case study
Source: PLOS Glob Public Health. 2023 Aug 24;3(8):e0002087. doi: 10.1371/journal.pgph.0002087 (PMC10449106; doi:10.1371/journal.pgph.0002087)
Supplement: S2 File — (DOCX) [file pgph.0002087.s002.docx]

**Script for semi-structured discussions with clinicians and potential assay users at field sites:**

**Interview Intro:** The goal of this discussion is to collect feedback on a rapid assay to detect slow-clearing partner drugs of artemisinin-based combination therapies from patient samples.  We seek your opinion on the utility of such an assay, realistic applications, and characteristics of the assay that will make it most effective.

This discussion is voluntary, for research purposes, and I appreciate your time. It should take 10-20 minutes and your feedback will be used to inform our design decisions about the assay. Please feel free to ask any clarifying questions throughout or to opt out of the discussion at any time. The discussion may be audio-taped if this is acceptable to you.

**Questions for health care workers/malaria researchers/funders:**

1. If health care worker:
   1. How frequently do you prescribe drugs for malaria?
   2. What drugs do you usually have in stock? What drugs do you have in stock right now?
   3. How do you decide which drugs to give?
   4. Do you ask or record what drugs patients have recently taken?
2. Are you concerned about the prevalence of fake/substandard drugs?
3. Are you aware of any initiatives/techniques to test for fake/substandard drugs?
4. Do you use malaria RDTs for your work?
5. Do you use any other point-of-care assays for your work?
   1. If so, which ones? Are they disposable? Bench top analyzers?
   2. What are some of the biggest challenges or frustrations to POC assay use in your clinic?
6. Would knowing which antimalarial drugs a patient has recently taken be useful do you?
7. Would you use a rapid test to detect antimalarial drugs from patient samples?
   1. How do you envision using such an assay?
   2. Elaborate on how it would be useful/not useful
8. Which drugs would be most helpful to detect and why?
9. If you had a rapid test, what is the easiest sample type to use and why?
   1. Finger stick blood?
   2. Venous blood?
   3. Urine?
   4. Saliva?
   5. Other?
10. If you had a rapid test, would you prefer a quantitative/semi-quantitative/or qualitative readout and why?
11. What other kinds of assay would be most useful to you in your clinic? i.e. better malaria RDT (improved sensitivity?), detecting fake/substandard drugs, identifying non-malarial fevers?

**Note on interview format:** Interviews were conducted one-on-one, in-person by author ESC (DPhil student).
